# Using the MMSE-2 to Measure Cognitive Deterioration in a Sample of Psychiatric Patients Living in Puerto Rico

**DOI:** 10.3390/ijerph18041694

**Published:** 2021-02-10

**Authors:** Sandra I. Ralat

**Affiliations:** Medical Sciences Campus, University of Puerto Rico, San Juan, PR 00936, USA; sandra.ralat@upr.edu

**Keywords:** cognitive deterioration, psychiatric patients, MMSE-2, Puerto Rico

## Abstract

Patients with psychiatric disorders often have cognitive impairment. Several deficits have been recognized in patients with mood and/or psychotic disorders. We hypothesized that differences in the levels of deterioration exist between patients with bipolar disorder (BD), major depressive disorder (MDD), and schizoaffective disorder (SAD). The mini-mental state examination, version 2 (MMSE-2), was used with a sample of 160 psychiatric patients to measure cognitive impairment. The aims of this study were as follows: (1) To characterize the differences in cognitive deterioration among patients diagnosed with BD, MDD, or SAD; (2) to explore item difficulty and cutoff points based on the educational level and other variables which are significant for our psychiatric population. Descriptive statistics were used for categorical variables. In addition, a Bonferroni post hoc test and an analysis of covariance (ANCOVA) for the continuous dependent variable were performed. Psychiatric diagnosis and years of education adjusted by several covariates proved to be significant. The 25th percentile were obtained to establish the cutoff points. Each item’s difficulty was analyzed using means and chi-square tests. Cognitive deterioration was found in 51% of the patients with SAD, in 31% with BD, and in 18% with MDD.

## 1. Introduction

Identifying cognitive impairment in psychiatric patients has an essential role in therapeutic interventions. To improve the usefulness of a given therapeutic intervention and, as a result, the overall functioning of the psychiatric patient with whom the intervention is to be employed, the level of that patient’s cognitive impairment must be determined. These cognitive deficits can be recognized early in the trajectories of these disorders. However, there are no consistent results on which domains are in deficits in patients with a psychiatric illness. Several studies have compared cognitive deterioration in psychiatric patients, indicating that these patients had comparable profiles [1]. Little difference has been found in performance in terms of memory and executive function for schizoaffective disorder (SAD) and bipolar disorder (BD) patients [2]. A significant overlap of impairment in the profiles of the neuropsychological tests of patients with mood disorders and of those with SAD has been found by other investigators [3]. Nevertheless, other studies found that patients with SAD exhibit a pattern of cognitive impairment which is similar to patterns exhibited by patients with schizophrenia, but distinct from those with major depressive disorder (MDD) or BD [4]. Patients with BD show neurocognitive impairment, with a large degree of heterogeneity among them [5]. In other words, SAD is distinct from mood disorders at the cognitive level [2]. In summary, some findings suggest that there are similarities between the neuropsychological test profiles of these patients [5], while others have found that patients with BD (without a history of psychosis) tend to have greater levels of cognitive dysfunction than do patients with MDD [6].

Cognition is the processing of information. Basic and higher levels of cognitive functions, such as attention, working memory, long-term memory, perception, decision making, executive control, and speech and language are all related to and affected by cognition [7]. A person who has trouble remembering, concentrating, learning, and/or making decisions that affect everyday life is said to have cognitive impairments [8]. By detecting cognitive deterioration, mental health workers could work toward enhancing both the psychosocial functioning and quality of life of these patients, both of which improvements are sound therapeutic clinical targets.

The mini-mental state examination (MMSE) is one of the most widely used screening tests for research. It is also used to make clinical assessments, with the aim of detecting cognitive impairment. This is a screening test, not a diagnostic test [9,10,11,12].

This screening test was originally developed by Folstein in 1975 [6]. In their 1987 study, Bird et al. [12] used the MMSE in a sample of Puerto Rican participants and found significantly higher cognitive impairment in that population than had been reported in US communities in that year [12]. The investigators also used the sample to study the impact of demographic variables together with other psychiatric diagnoses on MMSE scores. Possible explanations for the higher number of cases were that the Spanish version of the instrument may have yielded a greater number of errors; demographic variables, including the education level, may have affected the scores; or there may indeed have been a higher proportion of cognitively impaired people in PR than in the mainland.

Thirty-three years have passed since that study, and some of the items on the current translated (for the Hispanic community) version have changed. Several versions (and in different languages) have been developed and validated. In Puerto Rico, at the clinical level, the MMSE is used in conjunction with other diagnostic tests. We are not aware of any recent publications on this subject describing psychiatric patients in Puerto Rico. In searches through a number of open access journals, at least, nothing was found.

In different countries where the MMSE has been used, the question of cutoff points has arisen since there is no gold standard for this instrument. Several authors have written about the influence of sociodemographic variables (education, age, sex, etc.) over the cutoff score, but none can agree which is the most appropriate for detecting cognitive impairment [9,10]. For that reason, the use of different cutoffs, depending on years of education, is justified by the literature in order to decrease the number of false-positive results. For example, Crum et al. [10] recommended a cutoff score of 19 for persons with 0 to 4 years of education; a cutoff score of 27 for those with 9 to 12 years of education; and, finally, a cutoff score of 29 for those with a college education or higher, using the 25th percentile (lower quartile) of the MMSE score. Using different cutoff points was important since people with lower levels of education had lower scores on the MMSE, for that reason, it was necessary to adjust for the number of years of schooling. The standard cutoff score of 23 is the cut point which is still used worldwide (23 cases/24 non-cases) [11,13].

The aims of this study were as follows: (1) To characterize the differences in cognitive deterioration in patients with a diagnosis of BD, MDD, or SAD; (2) to explore item difficulty and the cutoff points based on educational and other variables which are significant for our population. In this study, the MMSE-2 was analyzed with a sample of patients to measure cognitive impairment. The research questions were “Can the MMSE-2 detect different levels of cognitive deterioration in these patients?” and “What are the possible cutoff points (adjusted by sociodemographic variables)?” I hypothesized that differences in the levels of deterioration for the different diagnosis categories exist and also that there would be different cutoff points for the instrument. Statistical methods for determining the cutoff points fall into two broad categories: Data oriented and results oriented [14]. Data-oriented methods are based on the calculation of quantiles, while results-oriented methods provide a cutoff value corresponding to the most significant relationship with a given result. They also assess the discriminative capacity of the diagnostic test, which is the ability of the test to differentiate healthy versus sick subjects [14]. Finally, the difficulty of the items on the MMSE-2 are presented (for the total sample and by diagnosis).

## 2. Materials and Methods

### 2.1. Participants

The data for the study described herein were drawn from another independent research study by the author [15]. We examined the MMSE-2 scores of 160 psychiatric patients who had been recruited from the Clinic of Albizu University in the San Juan area and from the outpatient and home-visit program of the Mental Health and Substance Abuse Administration. This cross-sectional study was approved by the institutional review board of the University of Puerto Rico, Medical Sciences Campus. The participants had BD, MDD, or SAD and ranged in age from 21 to 60 years.

A clinical psychologist or a social worker at each facility invited a given possible candidate to participate in the study. Only persons with a diagnosis of BD, MDD, or SAD were referred. After the initial approach, the PI was notified that she should contact the candidate. Before their enrollment in the study, potential participants provided a signed consent form. All the participants answered both a questionnaire that elicited sociodemographic information and verbal questions intended to gather mental and physical health data.

To meet the inclusion criteria, the participant had to have a diagnosis of BD, MDD, or SAD and had to be taking medication for his/her condition. The exclusion criteria were (the participant’s) having a substance abuse problem at the time of the interview or being in the midst of a suicidal crisis. Only 10% of the sample came from the private clinic of the academic institution. Of them, eight participants had BD and the other had MDD. The rest of the participants were referred from the outpatient sites and several homes where patients were living at the moment of the study. This was a convenience sample.

### 2.2. Instruments

The MMSE-2, Spanish version, assesses the cognitive status of an adult, using 11 domains (having a total of 30 items). These domains are registration, recall, orientation to time, orientation to place, attention and calculation, naming, repetition, comprehension, reading, writing, and drawing. This test can be used to screen for cognitive impairment. The maximum score that can be obtained is 30, where the score is indicative of no cognitive impairment. A score of 24 to 30 is considered normal [10,11]; 19 to 23 indicates mild cognitive impairment; 10 to 18, moderate cognitive impairment; and 0 to 9, severe cognitive impairment.

Baseline demographic measures were made, and the clinical characteristics of the subjects were determined (see Table 1). A statistical analysis was done using the IBM SPSS version 21 software. The internal consistency of the MMSE-2 was assessed by computing Cronbach’s alpha coefficient for this sample. We compared the MMSE-2 scores and sociodemographic variables of patients with BD, MDD, and SAD using several statistics. The statistical significance was set at α = 0.05. To review the cutoffs adjusted for education, the participants were subdivided into the same four age groups (referring to the educational level) which were used by Crum et al. [9] (see Table 2). Descriptive statistics were used for categorical variables. In addition, a Bonferroni post hoc test and an analysis of covariance (ANCOVA) for the continuous dependent variable were performed.

To establish the cutoff points, 25th percentiles were used, and item difficulty was analyzed using means and chi-square tests.

## 3. Results

### 3.1. Sample Characteristics

Forty-six percent of the participants had SAD; 32%, BD; and 22%, MDD. The mean age of the participants was 45.5 years (SD = 11.1 years; range, 21–60 years). Sixty-three percent were female. In regard to education, 6.3% had completed elementary school, only; 16.9% had completed middle school; 36% had completed high school; 11% had attended a technical college; 6% had an associate degree; 8.1% had a bachelor’s degree; 2.5% had a master’s degree; and 14.4% did not complete a university degree. The mean number of years of education was 11.9% (SD = 3.13 years). The marital status of the majority of the sample by diagnosis was single (χ^2^ = 25,750; *p* = 0.004). More detailed descriptions of this sample’s characteristics are reported in Table 1 (by diagnosis), with crosstabulation using chi-square analyses.

### 3.2. Statistics

For this sample, the MMSE-2 yielded a Cronbach’s alpha coefficient of 0.81. This is a very good level of reliability [16]. We conducted a one-way ANCOVA to analyze these data. Unadjusted means are presented, unless otherwise stated. The participants with MDD obtained a mean score of 24.03 (SD = 3.49; *n* = 35). BD participants obtained a mean score of 22.86 (SD = 4.0; *n* = 51). The participants with SAD obtained a mean score of 21.66 (SD = 3.95; *n* = 74). The MMSE-2 scores of the MDD participants were greater than those of the BDD and SAD participants.

Data are the adjusted mean ± standard error (SE), unless otherwise stated. The MMSE-2 scores were greater for the MDD participants (24.35 ± 0.60) than they were for both the BD participants (22.55 ± 0.48) and the SAD participants (21.73 ± 0.40).

After adjusting for education, gender, diet, and smoking, there was a statistically significant difference in the MMSE-2 scores between the diagnosis categories (*F*(2, 153) = 6.28; *p* = 0.002, η^2^ = 0.076). As expected, that specific difference in MMSE-2 scores observed between the diagnosis groups remains after adjusting by the covariates. By confirming that the MMSE-2 can in fact detect differing levels of cognitive deterioration in patients with BD, MDD, and SAD, these results provided us with an answer to our first question.

**Figure 1:**
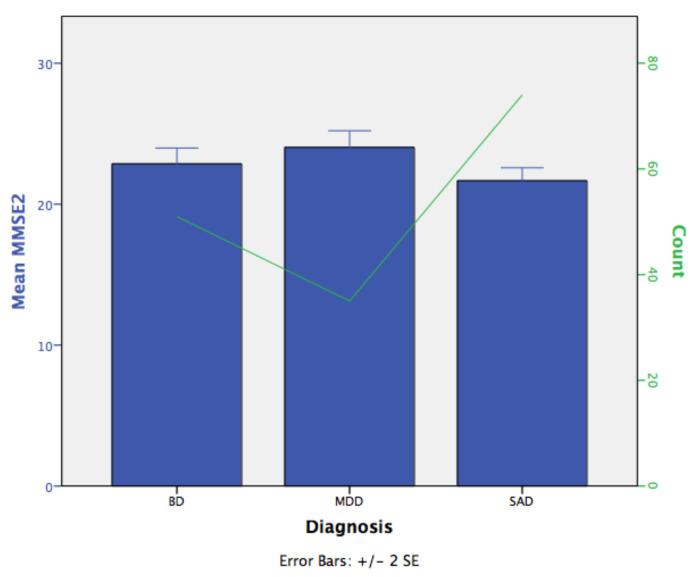
The standard errors (SEs) of the means of mini-mental state examination (MMSE-2) scores across the diagnosis categories.

Bonferroni post hoc comparisons examined the differences between groups (*p* < 0.05). MMSE-2 scores were statistically significantly greater in the MDD participants vs. the SAD participants (mean difference of 2.62 (95% CI, 0.828–4.41), *p* < 0.002). The differences between the MMSE-2 scores of the BD and MDD participants were not significant (mean difference of 1.79 (95% CI, −3.69–0.106), *p* < 0.071); neither were those between the BD and SAD participants (mean difference of 0.823 (95% CI, −0.673–2.32), *p* < 0.555). The participants with SAD had more cognitive impairment than did those with MDD. Figure 1 shows the SEs of the means of the MMSE-2 scores across the diagnosis categories. The number of cases by diagnosis is shown with the line that runs through the bars.

We found education to be a significant covariate. The results are the following: *F*(1, 153) = 45.78 (*p* = 0.000; η^2^ = 0.23).

Gender (*F*(1, 153) = 1.14; *p* = 0.288) and diet (*F*(1, 153) = 2.89; *p* = 0.091) were not significant covariates. However, smoking was a significant covariate, having an *F*(1, 153) = 4.02; *p* = 0.047; η^2^ = 0.026.

**Figure 2:**
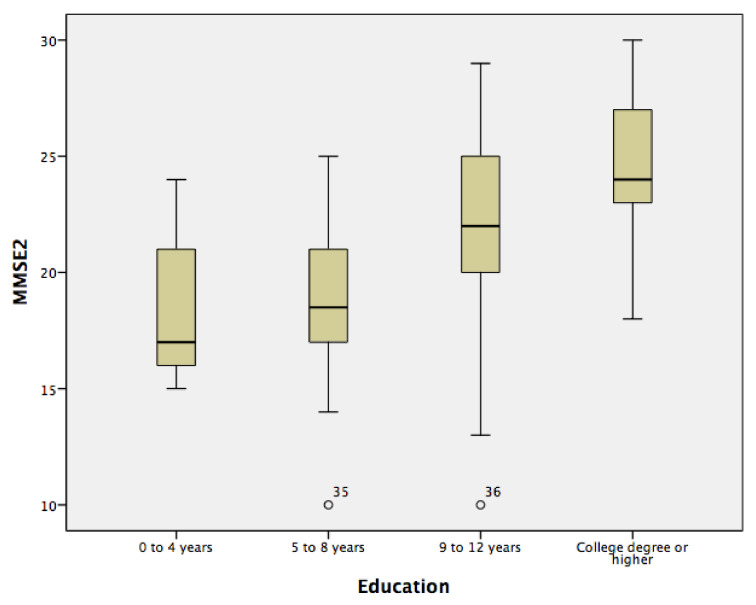
The distribution of MMSE-2 scores across years of education.

Another ANCOVA was performed, this one on the estimated MMSE-2 scores and with years of education as the independent variable and age as a covariate. The means and SDs are in Table 2. Data are the adjusted mean ± SE, unless otherwise stated. The MMSE-2 scores were greater in participants with a level of education reaching from college through a higher degree (13±) (24.48 ± 0.43) compared to individuals with 9 to 12 years of education (21.76 ± 0.41), 5 to 8 years of education (18.79 ± 0.94) or 0 to 4 years of education (18.72 ± 1.58). The more years of education a person had, the better his or her score on the MMSE-2 was, as previous literature has documented [10,11,12]. Figure 2 shows the distribution of the MMSE-2 scores across years of education.

Bonferroni post hoc comparisons examined differences between groups (*p* < 0.05). MMSE-2 scores were statistically significantly greater in the college degree or higher (13 ± years of education) participants vs. the participants with 0 to 4 years of education (mean difference of 5.76 (95% CI, 1.37–10.15), *p* < 0.004), the participants with 5 to 8 years (mean difference of 5.69 (95% CI, 2.93–8.44), *p* < 0.000), and the participants with 9 to 12 years (mean difference of 2.72 (95% CI, 1.13–4.30), *p* < 0.555).

After adjusting for age, there was a statistically significant difference in the MMSE-2 scores between the years of education, *F*(3, 155) = 15.22; *p* = 0.000, η^2^ = 0.23. Age as a covariate was not significant (*F*(1, 155) = 0.346; *p* = 0.557).

**Figure 3:**
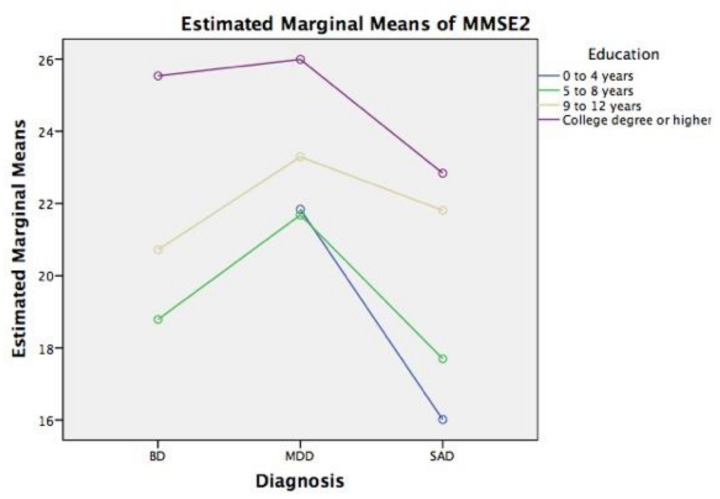
Estimated marginal means of MMSE-2 scores across the diagnosis and education categories, adjusting by smoking, diet, and gender, as covariates.

The Figure 3 below shows the estimated marginal means of the MMSE-2 scores across the independent variables and covariates.

After using the 25th percentile cutoff points as a reference, in order to decrease the number of false-positive results, we found the following: The 25th percentile for the lowest number of years of education (0–4 years) was around 16 (Table 2), which is similar to what was found by Bird et al., (1987). For the group possessing 5 to 8 years of education, the 25th percentile was 17, and it was 20 for the patients having 9 to 12 years of education. For the sample members having the highest levels of education, (13± years), the 25th percentile was 23. In summary, 64% of the participants were identified as not having any cognitive impairment, whereas 36% were found to have some degree of cognitive impairment.

According to the MMSE-2, looking at the entire sample, cognitive deterioration was found in 51% of the patients with SAD, 31% of those with BD, and 18% of those with MDD.

Regarding the MMSE-2 total scores (controlling for years of education and diagnosis), the distribution of normal vs. cognitive impairment (signified by a score of 23 or lower) was as follows: 59.5% of the SAD patients had scores less than or equal to 23; 52.9% of the BD patients and 45.7% of the MDD patients also had such scores (see Table 3).

### 3.3. Item Difficulty

Table 4 shows the means of item difficulty. We wanted to determine whether there were any differences in the responses of the participants by the diagnosis category, while also taking into consideration the item difficulty. These means were calculated by adding the scores of all the sample participants for a given item and dividing it by the number of scores [17]. The mean for each binary item is from 0 to 1. A mean close to or equal to 1 represents an easy item. A mean close to 0 represents a difficult item. Each item’s mean was calculated separately for the whole sample and by psychiatric diagnosis. A score of 1 indicates that all the participants answered that item (further indicating that the said item was a very easy one).

## 4. Discussion

The results revealed several key points. Generally, in a clinical or research setting, the MMSE-2 is used to compare a given patient’s score against the norms as they are found in the general population. Ideally, patient scores should be compared with the norms that apply to the population suffering from the same condition to assess how different such scores are from the scores of the members of that population [18]. In other words, for psychiatric patients, the examiner or clinician should interpret patients’ scores based on the cognitive performance of their peers. Unfortunately, in Puerto Rico, we do not have an established norm for psychiatric patients.

In my study, the sample exhibited differences in the levels of deterioration experienced by patients with BD, MDD, and SAD. Using the MMSE-2 to screen for cognitive impairment, with a score of 23 or less, we found that 51% of the participating SAD patients exhibited cognitive deterioration, as did 31% of the BD patients and 18% of the MDD patients. According to the results and the post hoc test, a significant result showed that participants with SAD had more cognitive impairment than did those with MDD. The participants with MDD had better scores. These results are important since they help identify the different levels of deterioration sustained by the members of each psychiatric group. This identification of cognitive impairment is essential for the design of psychosocial interventions in order to improve the functioning of these patients. However, it is recommended that this study be repeated, ensuring that the diagnosis categories have similar numbers of patients as well as a control group.

In this psychiatric sample, the MMSE-2 obtained a Cronbach’s alpha coefficient of 0.81, which is a very good reliability score.

Education was the principal variable with a significant effect on the MMSE-2 score, as we can see through the analysis of covariances. This finding is not different from those of other studies [19]. The result that differs from those of other studies is that, generally, age and other sociodemographic variables influence the MMSE-2 more or less the same as education does [19,20]. However, there are several studies that concur with this one [10,11]. In this case, the results agreed with others that also showed education to be a principal significant variable [21].

The 25th percentile provides important information regarding the cutoff points (with reference to the education variable). Table 2 displays the different cutoff points for the participants (based on years of education), using as a reference the study of Crum et al. [10]. The cutoff point of 16 was for participants with 0 to 4 years of education. For 5 to 8 years, the cutoff was 17; participants with 9 to 12 years of education had a cutoff point of 20, and those with 13± years, the cutoff was 23. These cutoff points do not concur with those of the Crum study, where the study used a different sample.

For a psychiatric patient, having a high level of education appears to improve the MMSE-2 scores. Contrary to expectations, this association did not hold true with SAD patients. The members of this group had lower scores than the BD and MDD patients did. Compared to the other two groups, the SAD patients were found to have worse cognitive functioning when we used a cutoff of 23, independently of education. That is significant information.

The present study revealed that there is a need for different cutoff points and that the educational level must be considered when cognitive deficits are being evaluated. However, it is also important to focus on how MMSE-2 scores are affected by different psychiatric diagnoses. 

We had to consider the different characteristics of the patients in three diagnostic groups as those differences relate to unhealthy lifestyle habits (smoking, no exercise, and high levels of stress). The majority of the participants (50% SAD and 31.4% BD) came from substitute homes, while 34.3% of the MDD participants had their own homes. Several medical comorbidities also were part of the clinical features of these participants as were weight-related health problems (see Table 1).

Finally, the items on the MMSE-2 that represented the greatest difficulty for the participants were those in the recall domain, specifically, the second and third words which were needed to be remembered. The items forming the attention and calculation domain (the second, third, fourth, and fifth calculation activities) and those of the repetition domain were also difficult for BD, MDD, and SAD patients to get through. For SAD patients, the most difficult items were recalling the second word given by the interviewer, and those having to do with attention and calculation. For BD patients, the most difficult item was repetition. The most difficult item for MDD patients was recalling word number three.

### Limitations

Several limitations of the study are worth noting. Only one screening test was used. It is recommended that an additional test—to determine the accuracy of the MMSE-2 scores of a specific patient—be deployed. The size of the diagnostic groups was not the same, as was the size of the group having the lowest number of years of education (0 through 8 years). A future study, one having larger sample sizes for each disorder and for the lower years of education, as well as a control group, should be undertaken (the inclusion of a healthy control group is essential). My study’s sample did not include any patients with schizophrenia. For that reason, I was unable to compare cognitive profiles or determine (as previous studies have done) whether there is any overlap in those profiles [5]. To determine whether having a high level of education affects the prevalence of either false positives or false negatives (by increasing them), studies that include sample members who have relatively low education levels need to be carried out [22]. Certain age and education levels had low numbers, and this may mean that, for these relationships, the findings are less reliable. In addition to the variables considered in this study, the cognitive functioning of the participants may have been influenced by other factors, such as the level of severity or degree of the psychiatric disturbance, social and cultural contexts, previous abilities, and the environment in which the assessment was carried out, as well as by the beginning of the symptomatology (which is, the number years with the diagnosis). This study is not representative of the entire population of Puerto Rico. All the participants had a diagnosis of BD, MDD, or SAD, though the psychiatric diagnosis could not be formally corroborated with a clinical interview.

Despite these limitations, the study draws attention to the different degrees of cognitive functioning in psychiatric patients, especially regarding these patients’ differing levels of education and the cutoff points used.

## 5. Conclusions

In conclusion, the current findings confirm that levels of cognitive deterioration in patients diagnosed with BD, MDD, or SAD were different. According to the MMSE-2 scores, patients with SAD had more cognitive impairment, independent of their having or not having high levels of education, than did those diagnosed with BD or MDD (or both). The 25th percentile can also be used when considering the different levels of education. The previous must be considered when the MMSE-2 is used with psychiatric patients, especially those who suffer from BD, MDD, or SAD. It is important to note that the variables of the education level and psychiatric diagnosis require distinct cutoff points. We were able to explore (by a diagnostic category) the items that the participants found difficult.

## Figures and Tables

**Table 1:** Sociodemographic characteristics and mental/physical health data.

Variable	BD		MDD		SAD		*χ* *^2^*	*p*-Value
Crosstabulation	%		%		%			
Gender	M	F	M	F	M	F	10.594	0.005
	41	59	14	86	46	54		
Age in years							21.675	0.154
21–24	3.9		2.9		5.4			
25–29	11.8		0.0		8.1			
30–34	5.9		5.7		12.2			
35–39	5.9		5.7		9.5			
40–44	21.6		11.4		12.2			
45–49	11.8		2.9		10.8			
50–54	15.7		28.6		23.0			
55–59	13.7		20.0		14.9			
60	9.8		22.9		4.1			
Education							4.146	0.657
0–4 years	0.0		5.7		4.1			
5–8 years	5.9		8.6		10.8			
9–12 years	45.1		45.7		45.9			
College or higher (13±)	49.0		40.0		39.2			
Residence							54.981	0.000
Own	15.7		34.3		1.4			
Rent	11.8		17.1		8.1			
Public housing	0		5.7		0			
Live with family	7.8		17.1		4.1			
Group home	15.7		0		13.5			
Long-term home	7.8		0		13.5			
Substitute home	31.4		11.4		50			
Transitional home	9.8		14.3		9.5			
Lifestyle								
Unhealthy diet	29.4		51.4		18.9		12.105	0.002
Exercise	52.9		60		48.6		1.234	0.540
Smoking	49		80		54.1		9.122	0.010
High levels of stress	49		80		54.1		9.122	0.010
Medical comorbidities								
Hypertension	35.3		48.6		29.7		3.682	0.159
Hypothyroidism	28.6		25.7		31		0.324	0.851
Asthma	20.4		20.0		21.1		0.021	0.990
Diabetes	21.6		25.7		13.5		5.338	0.254
Hypoglycemia	0		5.7		4.1			
High cholesterol	27.5		28.6		31.1		0.206	0.902
Weight-related health problem							5.517	0.479
Obesity BMI > 30 kg^2^	43.1		45.7		45.9			
Overweight	33.3		42.9		32.4			
Underweight	0		2.9		4.1			
Non-obesity	23.5		8.6		17.6			
Number of medications							29.783	0.003
One	0		3		0			
Two	2		5.7		2.7			
Three	13.7		11.4		4.1			
Four to six	47.1		45.7		41.9			
Seven to nine	15.7		5.7		18.9			
10 or more	19.6		8.6		29.7			

**Table 2:** The 25th percentiles are the cutoff points for each educational level (to decrease the number of false-positive results).

		Years of Education			
		0–4 Years	5–8 Years	9–12 Years	13± Years
N	Cases	5	14	73	68
Mean		18.60	18.79	21.75	24.50
Median		17.00	18.50	22.00	24.00
Standard deviation		3.782	3.906	3.778	3.059
Range		9	15	19	12
Percentiles	25	16	17	20	23
	50	17.00	18.50	22.00	24.00
	75	22.50	21.25	25.00	27.00

**Table 3:** MMSE-2 score by the number of years of education and diagnosis.

Diagnosis			MMSE-2 Results		Total (%)
			Normal	Cognitive Impairment	
			(>24 Score)	(<23)	
BD	Years of education	5–8	0	3	3 (6%)
		9–12	6	17	23 (45%)
		13± (college and higher)	18	7	25 (49%)
	Total		24 (47.1%)	27 (52.9%)	51 (100%)
MDD	Years of education	0–4	1	1	2 (6%)
		5–8	1	2	3 (8%)
		9–12	7	9	16 (46%)
		13± (college and higher)	10	4	14 (40%)
	Total		19 (54.3%)	16 (45.7%)	35 (100%)
SAD	Years of education	0–4	0	3	3 (4%)
		5–8	1	7	8 (11%)
		9–12	15	19	34 (46%)
		13± (college and higher)	14	15	29 (39%)
	Total		30 (40.5%)	44 (59.5%)	74 (100%)
Total	Years of education	0–4	1	4	5 (3%)
		5–8	2	12	14 (9%)
		9–12	28	45	73 (46%)
		13± (college and higher)	42	26	68 (42%)
	Total		73 (45.6%)	87 (54.4%)	160 (100%)

**Table 4:** Item difficulty: Total sample and by diagnosis.

MMSE-2 Items	Mean, All	Mean, BD	Mean, MDD	Mean, SAD
Registration				
Word 1	1.00	1.00	1.00	1.00
Word 2	0.97	0.98	0.97	0.96
Word 3	0.92	0.90	0.94	0.92
Orientation to time				
Year	0.94	0.96	0.97	0.91
Season	0.68	0.59	0.66	0.74
Month	0.91	0.94	1.00	0.85
Day	0.90	0.92	1.00	0.84
Date	0.78	0.82	0.94	0.68
Orientation to place				
State	0.98	1.00	1.00	0.96
City	0.94	0.94	1.00	0.91
Neighborhood/Community	0.73	0.65	0.74	0.78
Place	0.90	0.92	0.89	0.89
Floor	0.90	0.82	0.94	0.93
Recall				
Recall word 1	0.85	0.86	0.91	0.81
Recall word 2	0.38	0.39	0.54	0.3 ^a,1^
Recall word 3	0.26	0.27	0.2 ^b^	0.28
Attention and calculation				
Calculating 1	0.53	0.61	0.6	0.45
Calculating 2	0.26	0.27	0.37	0.2 ^a^
Calculating 3	0.33	0.39	0.43	0.23 ^a,2^
Calculating 4	0.34	0.45	0.49	0.2 ^a,3^
Calculating 5	0.32	0.35	0.43	0.24 ^a^
Naming				
Naming1	0.99	0.98	1.00	0.99
Naming2	1.00	1.00	1.00	1.00
Repetition				
Repetition	0.28	0.25 ^c^	0.34	0.27
Comprehension				
Comprehension1	0.98	0.98	0.97	0.99
Comprehension2	0.97	0.98	0.97	0.96
Comprehension3	0.97	1.00	0.97	0.95
Reading				
Reading	0.99	1.00	1.00	0.97
Writing				
Writing	0.92	0.94	1.00	0.86
Drawing				
Drawing	0.65	0.67	0.74	0.59

The most difficult items for the sample were the second ^a^ (especially for schizoaffective disorder (SAD) patients) and third ^b^ (especially for major depressive disorder (MDD)) words that had to be remembered. Activities number two ^a^, three ^a^, four ^a^, and five ^a^ of the attention and calculation domain (especially for SAD patients); and repetition ^c^ (especially for bipolar disorder (BD) patients). ^1^ χ^2^ = 6.112; *p* = 0.047 (two-sided, 0.05 significance); ^2^ χ^2^ = 5.822; *p* = 0.027 (two-sided, 0.05 significance); ^3^ χ^2^ = 12.252; *p* = 0.001 (two-sided, 0.05 significance).

## Data Availability

Not applicable.
